# Application of Hansen Solubility Parameters in the Aqueous-Ethanol Extraction of Genistein-7-O-[α-rhamnopyranosyl-(1→6)]-β-glucopyranoside from *Derris scandens* and Its Molecular Orbital Study on Antioxidant Activity

**DOI:** 10.3390/ijms26125740

**Published:** 2025-06-15

**Authors:** Thitiporn Tantinithiphong, Wanna Eiamart, Sarin Tadtong, Suwanna Vorarat, Weerasak Samee

**Affiliations:** 1 Department of Pharmaceutical Chemistry, Faculty of Pharmacy, Srinakharinwirot University, Nakhon Nayok 26120, Thailand; thitiporn.tan@g.swu.ac.th (T.T.); suwannav@g.swu.ac.th (S.V.); 2 Technical and Planning Division, Department of Thai Traditional and Alternative Medicine, Ministry of Public Health, Nonthaburi 11000, Thailand; 3 Chula Pharmacokinetic Research Center, Faculty of Medicine, Chulalongkorn University, Bangkok 10330, Thailand; wanna.e@chula.ac.th; 4 Department of Pharmacognosy, Faculty of Pharmacy, Srinakharinwirot University, Nakhon Nayok 26120, Thailand; sarin@g.swu.ac.th

**Keywords:** solvent selection, flavonoid, polyphenol, chromatography, validation, green solvent

## Abstract

This study explored the extraction of genistein-7-O-[α-rhamnopyranosyl-(1→6)]-β-glucopyranoside (GTG) from *Derris scandens* using an aqueous-ethanol solvent system, aiming to optimize yield and antioxidant activity. Hansen solubility parameters (HSP) were employed to determine the optimal solvent composition, with the highest GTG yield (6.83 ± 0.06 mg/g dried weight) obtained from 50% ethanol—correlating well with HSP predictions. Ultrasonic extraction was most effective with solvents having a dielectric constant between 50 and 60. The antioxidant potential of isolated GTG was evaluated using the DPPH assay, which yielded an IC_50_ of 87.86 ± 1.85 μM, and the FRAP assay, with a value of 34.23 ± 2.75 mg FeSO_4_ equivalents. Molecular orbital analysis revealed HOMO and LUMO energy gaps (ΔE = 10.6715 eV) similar to known antioxidants such as gallic acid, ascorbic acid, Trolox, and quercetin. These findings demonstrate that HSP effectively guided solvent selection for ultrasound-assisted extraction of GTG. The antioxidant activity is attributed to GTG’s capacity to donate electrons and stabilize radicals via extended charge delocalization within the aglycone structure, confirming its potential as a natural antioxidant agent.

## 1. Introduction

**Figure 1:**
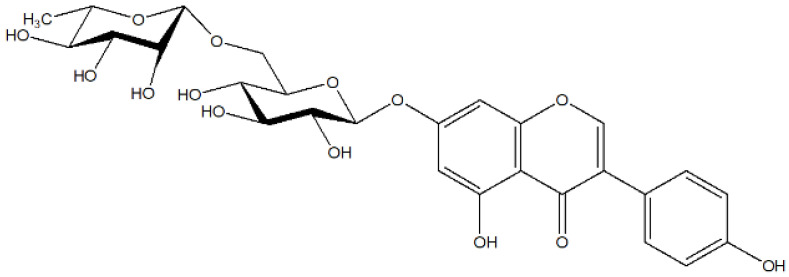
Chemical structure of GTG.

*Derris scandens*, recognized for its traditional medicinal applications, has garnered considerable interest due to its abundant flavonoid content, which plays a significant role in its biological activity. Flavonoids are polyphenolic compounds that exhibit a wide array of bioactivities, including antioxidants, anti-inflammatory, antibacterial, and anticancer properties [1,2,3,4,5,6]. Genistein-7-O-α-rhamnopyranosyl-(1→6)-β-glucopyranoside (GTG) is a significant isoflavonoid compound isolated from *Derris scandens*, recognized for its potential health benefits. As depicted in Figure 1, GTG belongs to the class of glycosylated isoflavones, which are characterized by a wide range of biological activities, including antioxidants, anti-inflammatory, and estrogenic effects [7,8,9]. Furthermore, research suggests that structural modifications of genistein can augment its bioactivity; notably, glycosylation at the 7-O position with α-rhamnopyranosyl and β-glucopyranosyl groups may enhance its stability, solubility, and, consequently, its bioavailability compared to the aglycone form of genistein [10,11,12]. Moreover, research has indicated the presence of multiple phenolic compounds in *D. scandens* that contribute to its radical scavenging abilities, thereby imparting antioxidant effects [13]. The extraction of GTG from *D. scandens* commonly utilizes ethanol or methanol as solvents, owing to their effectiveness in dissolving various phytochemicals. Research has demonstrated that extracts of *D. scandens* can be optimized through methods such as ultrasonic-assisted extraction or microwave-assisted extraction, which may enhance the yield of bioactive constituents, including GTG [14,15,16].

The dielectric constant is a fundamental parameter in materials science and electrical engineering, reflecting a material’s capacity to store electrical energy within an electric field. It measures the extent to which a material can become polarized when subjected to an electric field, providing critical insights into the solubility behavior of polar and non-polar substances in various solvents. Materials with high dielectric constants are typically polar, enabling interactions such as dipole-dipole and hydrogen bonding, whereas those with low dielectric constants are generally non-polar, characterized by weaker London dispersion forces [17]. The relationship between dielectric constants and the extraction of phytochemicals is pivotal in the fields of natural product chemistry and pharmacognosy. The dielectric constant of solvents is crucial in determining their effectiveness in dissolving and extracting phytochemicals due to the polarity of the compounds involved. Solvents with high dielectric constants are typically more effective at solubilizing polar compounds, whereas solvents with low dielectric constants are preferred for extracting nonpolar substances [18,19,20,21,22]. Thus, selecting appropriate solvents based on their dielectric properties is essential for improving the yield and efficacy of phytochemical extracts.

The Hansen solubility parameter (HSP) is a fundamental concept for understanding the extraction of flavonoids and other phytochemicals. This parameter categorizes solubility into three components: dispersion forces (δd), polar forces (δp), and hydrogen bonding (δh), collectively assessing a solvent’s ability to dissolve various solutes based on their interactions [23,24,25]. This comprehensive approach allows researchers to identify appropriate solvents for extracting bioactive compounds, such as flavonoids, which exhibit varying degrees of polarity and hydrogen bonding capabilities [25,26]. This principle of aligning solvents with solutes is further emphasized by the work of Novaes et al., who describe how HSPs facilitate the selection process for efficient extraction methods, thereby minimizing solvent wastage and enhancing overall yield [25]. Furthermore, Zhang et al. corroborated that HSPs can significantly influence the development of eco-friendly solvents, underscoring their importance in the context of green chemistry and sustainable practices in extraction processes [27].

Ultrasonic-assisted extraction (UAE) is a highly effective technique for recovering flavonoid glycosides from various plant materials. This method leverages ultrasonic waves to enhance extraction efficiency by facilitating solvent penetration into plant tissues, leading to the breakdown of cell walls and aiding in the release of intracellular components, including flavonoids [28,29,30]. The relationship between the extraction method and the dielectric properties of the solvents used is fundamental in optimizing the process and ensuring high yield and quality of extracted flavonoids. The mechanism of ultrasonic extraction is grounded in the generation of cavitation bubbles in the solvent, which collapse and create shock waves. This physical phenomenon enhances the mass transfer of flavonoids from the plant matrix into the solvent [31]. Moreover, the choice of solvent in UAE significantly influences the dielectric constant, which in turn affects solubility and extraction efficiency [32,33,34].

A critical component in evaluating the efficacy of antioxidant compounds is deeply rooted in the principles of frontier molecular orbital (FMO) theory, specifically focusing on the energies of the highest occupied molecular orbital (HOMO) and the lowest unoccupied molecular orbital (LUMO). The antioxidant activity of various compounds is intrinsically linked to their electronic properties, as illustrated by the HOMO–LUMO energy gap; notably, a narrower gap often indicates increased reactivity, which can enhance antioxidant potential [35,36]. This dynamic was further explored in studies on flavonoids, where specific molecular structures correlated with elevated radical scavenging abilities due to favorable electron transfer characteristics [37,38]. FMO theory is integral to this mechanism; the HOMO facilitates electron donation, while the LUMO enables electron acceptance, thereby promoting charge transfer and enhancing antioxidant activity [39]. Studies have shown that the presence of hydroxyl groups in certain structures improves electron-donating capacity, primarily influenced by their HOMO energy levels [40,41,42,43]. Additionally, the degree of electron delocalization throughout a molecular structure significantly impacts its stability and, consequently, its antioxidant capacity. Compounds exhibiting extensive charge delocalization within FMO enhance the stabilization of radical species, thereby increasing their radical scavenging effectiveness [44,45]. The intersection of frontier molecular orbital theory and antioxidant chemistry underscores the critical role of electronic properties in determining the activity of antioxidant compounds. The correlation between HOMO and LUMO energies and antioxidant effectiveness provides a framework for the strategic development of new therapeutic agents aimed at combating oxidative stress-related diseases.

This study focused on the efficacy of the solvent extraction method aimed at maximizing the yields of GTG through optimal solvent selection, specifically utilizing eco-friendly solvents such as ethanol and water, based on their HSP and dielectric constants. The GTG content across various solvent ratios was quantitatively analyzed using reverse-phase high-performance liquid chromatography (HPLC) with UV detection at 260 nm. Additionally, the total phenolic and total flavonoid contents of the extracts were assessed. The antioxidant activity of the extracts was evaluated using DPPH and FRAP assays. The HOMO and LUMO of GTG were also calculated and compared with reference antioxidants such as gallic acid, genistein, Trolox, and quercetin. The study provides a detailed discussion on the relationships among HSP parameters, dielectric constants, phytochemical contents, and antioxidant activity, providing valuable insights into extraction optimization and the underlying mechanisms of antioxidant action via frontier molecular orbital analysis.

## 2. Results

### 2.1. Preparation of In-House GTG from Derris scandens

**Figure 2:**
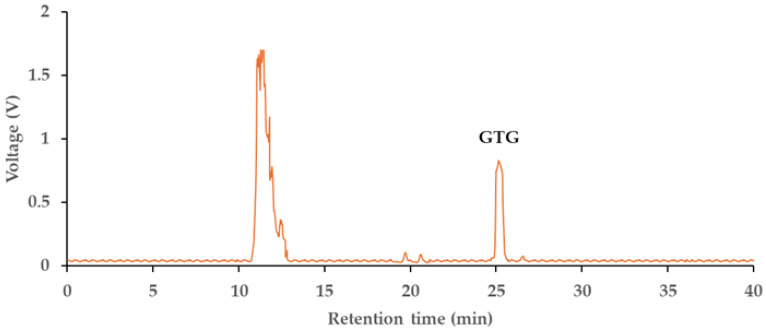
Semi-preparative HPLC chromatogram of the aqueous fraction of *Derris scandens* water extract, detected at 260 nm.

Since GTG was not commercially available in the market, studies on GTG required extraction and separation from herbs that contained a high amount of GTG. In previous research, GTG was identified as the principal compound in the aqueous extract of *D. scandens* [46]. In this study, GTG was extracted from the stem powder of *D. scandens* using water as the solvent. Ethyl acetate was employed to partition the non-polar compounds from the aqueous extract. The aqueous fractions were subsequently evaporated and subjected to semi-preparative HPLC for the separation of GTG from the mixture. The semi-preparative chromatogram is presented in Figure 2. The mobile phase eluted between 24.8 and 26.2 min was collected and evaporated to prepare the in-house GTG for subsequent analyses.

### 2.2. Identification of GTG in Derris scandens Extracts

**Figure 3:**
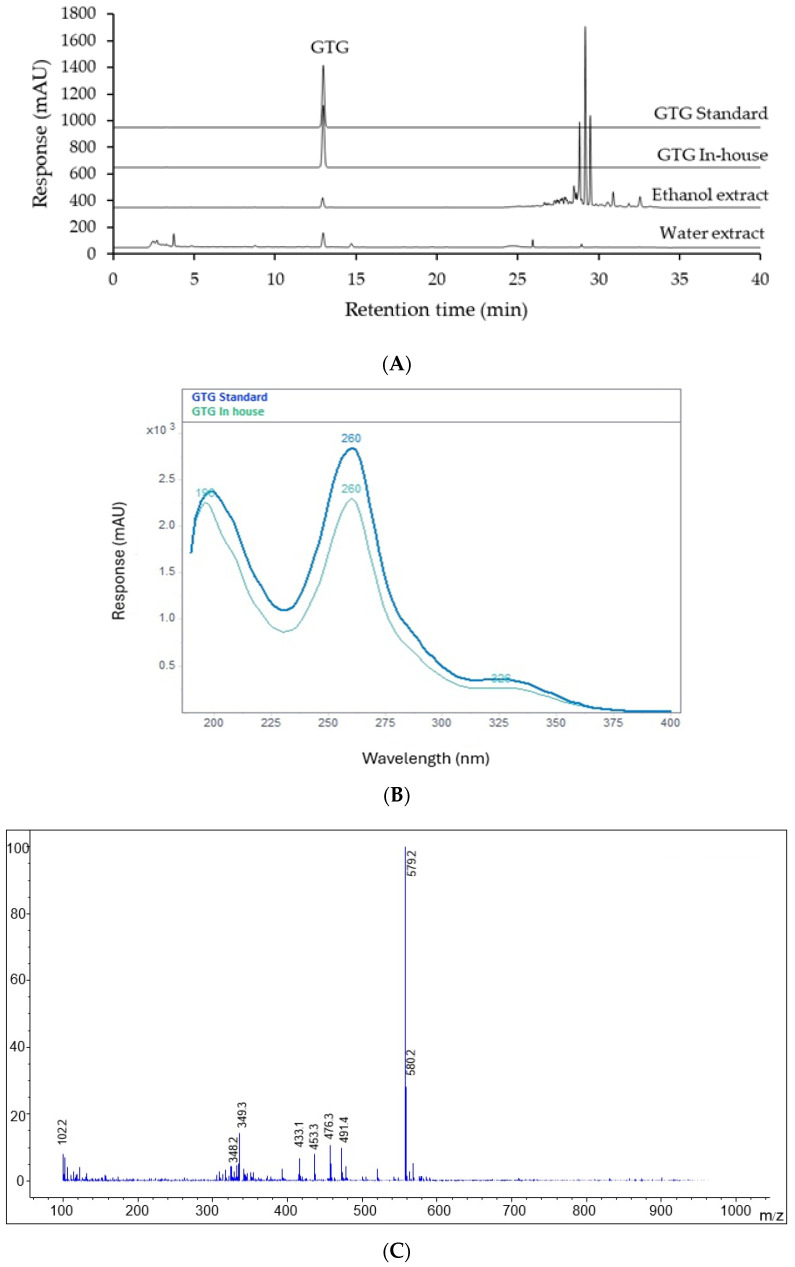
Characterization of the in-house GTG compound via (**A**) high-performance liquid chromatography (HPLC) retention time comparison with a reference GTG standard; (**B**) UV-absorption spectrum comparison with the reference GTG standard; and (**C**) mass spectrum analysis of the in-house GTG in positive ionization mode.

To accurately identify the in-house GTG and GTG in *D. scandens* samples, multiple analytical methods, including high-performance liquid chromatography with photodiode array detection (HPLC-PDA), ultraviolet (UV) spectroscopy, and liquid chromatography-mass spectrometry (LC-MS), were employed. The HPLC-PDA analysis, presented in Figure 3A, demonstrated that the GTG peak of the in-house standard co-eluted with the GTG standard, all possessing a retention time of 12.95 min. The UV spectra, shown in Figure 3B, exhibited identical absorption patterns for both the in-house and standard GTG, with a maximum absorption wavelength (λmax) at 260 nm. LC-MS analysis detected a fragment ion at *m*/*z* 579.2 in positive ion mode (Figure 3C), corresponding to the molecular weight of GTG (578 Da), as reported by Sae-Foo et al. [46].

### 2.3. Validation of HPLC Analytical Method for Determination of GTG in Derris scandens Extracts

**Figure 4:**
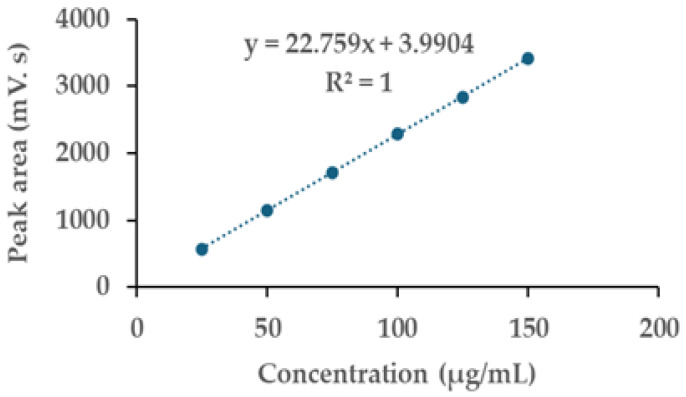
Calibration curve for GTG.

The analytical method for GTG analysis in *D. scandens* extracts was developed as detailed in Section 4.4. The retention time for GTG was approximately 12.95 min, as demonstrated in Figure 3A The analytical method was subjected to both system suitability testing and method validation to ensure compliance with the standards established by the International Council for Harmonisation (ICH) for enhanced healthcare [47], as well as the AOAC Guideline 2016 [48]. The analysis was performed on a 20% ethanol extract of *D. scandens*, conducted six times under specified high-performance liquid chromatography (HPLC) conditions, using an injection volume of 10 µL. The system suitability for the GTG was confirmed to be within acceptable limits, evidenced by a percentage relative standard deviation (% RSD) of retention time and peak area that was less than 2%. Each injection produced theoretical plates exceeding 2500, with a tailing factor maintained at less than ±1.01. The count of theoretical plates serves as a direct measure of a chromatographic column’s efficiency. A higher number of theoretical plates indicates a more effective separation, resulting in sharper and more well-defined peaks during analyte elution. Additionally, the resolution obtained was greater than 2.0. These findings confirmed that the method satisfied the acceptance criteria outlined in the ICH guidelines, as shown in Table 1. The details of the method validation results are presented in Table 2 as follows:

Linearity: the calibration curve for GTG demonstrated a linear relationship within the concentration range of 25 to 150 µg/mL, achieving a correlation coefficient of 1 (Figure 4), thereby satisfying the requirement of being greater than 0.995.

Limit of detection (LOD) and limit of quantitation (LOQ): From the three linear equations analyzed, the standard deviation of the y-intercept was found to be 2.690, while the average slope of the linear equations was 22.759. As a result, the concentrations for the LOD and LOQ were established at 0.39 µg/mL and 1.18 µg/mL, respectively.

Accuracy: The mean percentage recovery of the GTG standard solution at concentrations of 40, 80, and 120 µg/mL were found to be 98.90%, 99.21%, and 99.10%, respectively. All recovery values fell within the acceptable range of 95% to 105% as established by the AOAC Guideline 2016.

Precision: The findings showed that the percentage relative standard deviation (%RSD) for intra-day repeatability ranged between 0.48% and 1.93%, while the %RSD for inter-day intermediate precision ranged from 0.76% to 1.30%. Both values fell within the acceptable threshold of less than 3.7%, as specified in the AOAC Guideline 2016. The results of the method validation demonstrated that the method conformed to the acceptance criteria outlined in the AOAC Guideline 2016, as detailed in Table 2.

### 2.4. Solvent Selection for the Extraction of GTG from Derris scandens

The extraction of GTG from *D. scandens* necessitates the careful selection of solvents to optimize yield while minimizing toxicity for oral administration, as well as considering environmental and sustainability factors. Several factors influence this selection process, including the polarity of GTG, the efficiency of the extraction methods employed, and the chemical properties of potential solvents. For the extraction process, various ratios of ethanol and water were utilized, employing the ultrasonic extraction method. The Hansen solubility parameter provides a comprehensive model that considers three intermolecular forces—dispersion forces (δd), polarity (δp), and hydrogen bonding (δh)—which collectively describe a solvent’s ability to dissolve GTG based on its molecular characteristics. As shown in Table 3, the calculated values for δd, δp, and δh of GTG were 19.8610, 12.2874, and 35.5022, respectively.

The distance (Ra) between the Hansen solubility parameters of a solute and solvent is a critical factor in the solvent selection process for GTG extraction from *D. scandens*. This measurement provides insights into the compatibility between the solute and the solvent based on their respective dispersion, polar, and hydrogen bonding parameters. Lower Ra values indicate higher miscibility and stronger molecular interactions between the selected solvents [23]. As presented in Table 4, the Ra values for GTG in relation to water and ethanol were 19.45 and 20.01, respectively. The Ra values for mixed solvents were lower than those for single solvents, with values of 17.46, 15.91, 15.60, 15.74, and 16.98 for 80% ethanol, 60% ethanol, 50% ethanol, 40% ethanol, and 20% ethanol, respectively. These results suggest that GTG demonstrates the greatest solubility in 50% ethanol, which can be attributed to the lowest Ra values associated with these solvent mixtures.

### 2.5. The Relationship of Extracted GTG Content to Hansen Solubility Parameters and Dielectric Constants

**Figure 5:**
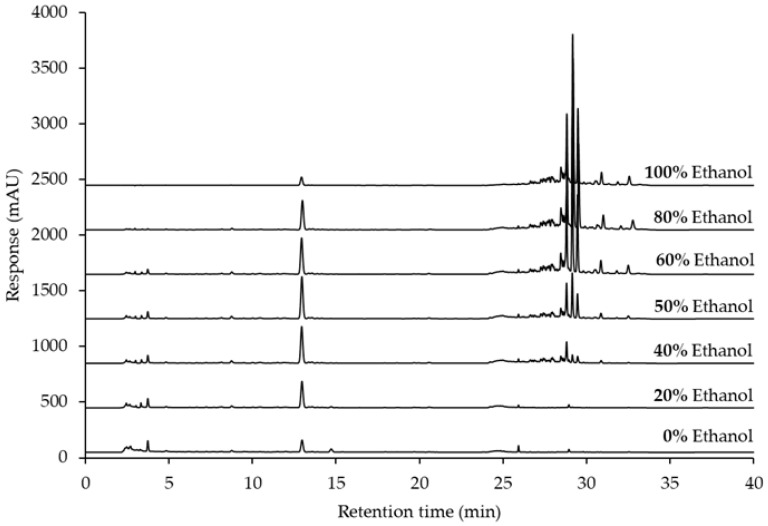
Chromatograms of aqueous-ethanolic extracts of *Derris scandens*, detected at 260 nm using UV detection.

Ultrasonic-assisted extraction (UAE) utilizing aqueous-ethanol solvent systems has emerged as a highly effective method for extracting bioactive compounds, particularly flavonoids such as GTG, from plant materials. Methodologically, UAE enhances extraction kinetics through the generation of cavitation bubbles, which disrupt plant cell walls and facilitate the efficient solubilization and extraction of GTG [49]. This extraction technique harnesses the synergistic effects of ultrasound combined with optimal solvent ratios to improve both yield and efficiency. The extraction of GTG from *D. scandens* using aqueous-ethanol solutions has been systematically investigated to augment GTG content. In this study, various ratios of water and ethanol were utilized as extractive solvents for UAE. As depicted in Figure 3A and Figure 5, the retention time for GTG was approximately 12.95 min, with small peaks of polar compounds detected at retention times below 5 min in extracts containing 0% to 60% ethanol. Conversely, non-polar compounds were identified at retention times exceeding 25 min in extracts ranging from 40% to 100% ethanol. Sukhonthasilakun et al. (2023) reported that the extraction with 95% ethanol has been found to produce higher quantities of lupalbigenin alongside other isoflavones [50].

According to the data presented in Table 5, the highest concentration of GTG was observed in the 50% ethanol extract, while the lowest concentration was recorded in the pure ethanolic extract. These findings indicate that aqueous-ethanol solvents are well-suited for the extraction of GTG from *D. scandens*. The application of UAE in combination with aqueous-ethanol solvent systems represents a highly effective approach for the extraction of GTG from *D. scandens*.

**Figure 6:**
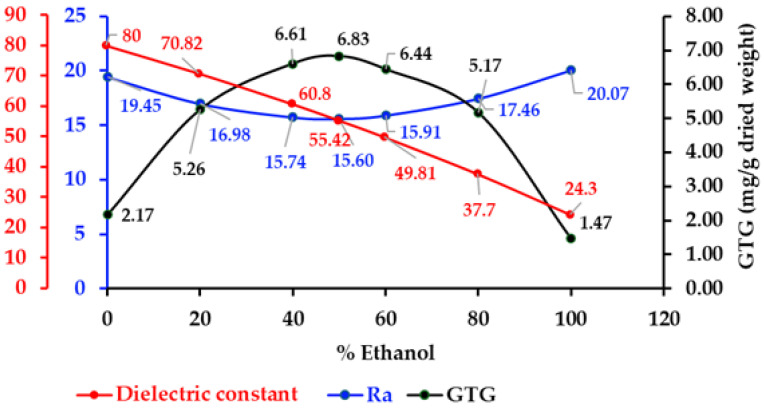
The relationship between extracted GTG content, Hansen solubility parameters, and dielectric constants.

The quantitative relationship between GTG content and the radius of the Hansen solubility parameters (Ra) was grounded in the principle that solubility was influenced by the degree of similarity between the solubility parameters of the solute and the solvent system. In this context, the Hansen solubility parameters—comprising dispersion (δd), polar (δp), and hydrogen-bonding (δh) components—provided a three-dimensional framework for predicting solubility. The choice of an aqueous-ethanol solvent system was motivated by the objective of minimizing Ra. An optimal solvent exhibited Hansen solubility parameters that closely aligned with the target values for GTG. As presented in Table 4 and Figure 6, the GTG extraction contents demonstrated a correlation with lower Ra values. The lowest Ra of 50% ethanol (15.60) was associated with the highest GTG content in the 50% ethanolic extract (6.83 ± 0.06 mg/g dried weight). Conversely, the highest Ra for ethanol (20.07) corresponded with the lowest GTG content in the ethanolic extract (1.47 ± 0.02 mg/g dried weight). These findings suggested that Hansen solubility parameters served as an effective tool for enhancing GTG extraction from *D. scandens*.

The dielectric constant of the aqueous-ethanol solvent system, as observed in Table 5 and Figure 6, indicates the highest yield of GTG within the dielectric constant range of 50 to 60, corresponding to ethanol concentrations between 40% and 60%. GTG, which possesses multiple hydroxyl groups and an aglycone nucleus, exhibits a polarity that enables it to participate in both polar and non-polar interactions with the solvent. In mixed solvent systems, the overall dielectric constant is influenced by the ratio of ethanol to water; water has a high dielectric constant of approximately 80, whereas ethanol’s dielectric constant is significantly lower at around 24.30. This results in an intermediate dielectric constant in aqueous-ethanol mixtures, which can be fine-tuned to closely match the polarity of GTG, thereby enhancing solubilization and extraction efficiency.

### 2.6. Total Phenolics, Total Flavonoids and Antioxidants Activities of Derris scandens Extracts

The data summarized in Table 6 provide a comprehensive evaluation of the total phenolic content (TPC), expressed as milligrams of gallic acid equivalents per gram of dried *D. scandens* powder (mg GAE/g), utilizing the calibration equation Y = 0.064X + 0.0115 (R^2^ = 0.9999). The total flavonoid content (TFC) was measured as milligrams of quercetin equivalents per gram of dried *D. scandens* powder (mg QE/g), based on the calibration equation Y = 0.011X + 0.0061 (R^2^ = 0.9999). The results demonstrated that the phenolic content across the extracts ranged from 17.46 ± 1.65 to 31.53 ± 0.61 mg GAE/g, with the highest concentration observed in the 50% ethanolic extract. Similarly, flavonoid content varied from 12.42 ± 0.40 to 44.25 ± 1.08 mg QE/g, with the maximum flavonoid content detected in the 100% ethanolic extract. The 50% ethanol extract exhibited the highest total phenolic content (TPC), which was positively correlated with the heat shock parameters (HSP) of GTG. The correlation between TPC and GTG suggests that GTG is the predominant phenolic acid extracted in aqueous-ethanol solvents. In contrast, the 100% ethanol extract showed the highest total flavonoid content (TFC). This could be attributed to the influence of aglycone flavonoids, such as lupalbigenin [50], which are more soluble in the lower polar solvent (ethanol) and did not demonstrate a correlation with the HSP of GTG.

The DPPH (2,2-diphenyl-1-picrylhydrazyl) assay is a widely used method to evaluate the free radical scavenging capacity of a substance. When an antioxidant is present, it donates electrons or hydrogen atoms to DPPH radicals, neutralizing them and causing a color change from purple to yellow. The antioxidant activity was expressed as the 50% inhibitory concentration (IC_50_), representing the sample concentration required to inhibit 50% of DPPH radicals. The Ferric Reducing Antioxidant Power (FRAP) assay measures the ability of the sample to reduce ferric ions (Fe^3+^) to ferrous ions (Fe^2+^). The assay results are expressed as the concentration of FeSO_4_ equivalent, with higher absorbance indicating greater reducing (antioxidant) capacity. Spectrophotometric measurement of absorbance reflects the electron-donating ability of the sample, which correlates with its antioxidant potential.

**Figure 7:**
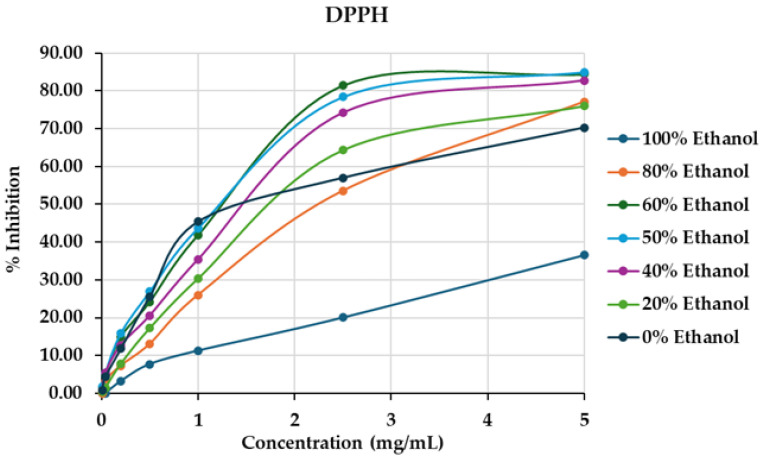
The relationship between the concentrations of extracts obtained using various solvents and their % inhibition in the DPPH assay.

As presented in Figure 7 and Table 6, the water extract exhibited the highest DPPH antioxidant activity, with an IC_50_ value of 1.08 ± 0.01 mg/mL. Conversely, the ethanol extract demonstrated the lowest activity, with an IC_50_ that could not be determined, as the percentage inhibition did not reach 50%. In the FRAP assay, the water extract also showed the highest reducing power, with a value of 762.46 ± 47.95 µg/mL FeSO_4_/g dried weight, while the ethanol extract exhibited the lowest FRAP value (253.04 ± 3.47 µg/mL FeSO_4_/g dried weight). The DPPH scavenging activities of the aqueous-ethanol mixed solvent extracts were comparable, with IC_50_ values ranging from 1.41 ± 0.01 to 2.30 ± 0.02 mg/mL. Similarly, the FRAP values ranged from 422.16 ± 34.05 to 550.32 ± 30.71 mg FeSO_4_/g dried weight. No significant correlation was observed between antioxidant activities and the levels of total phenolics or total flavonoids. These results suggest that antioxidant compounds from *D. scandens* are primarily extracted using polar solvents.

The apparent discrepancy between the highest antioxidant activity (AOA) observed in the water extract and the peak total phenolic content (TPC) in the 50% ethanol extract, as well as the highest total flavonoid content (TFC) in the 100% ethanol extract, suggests that compounds beyond phenolics and flavonoids may substantially contribute to the antioxidant capacity. Water extracts are known to contain water-soluble antioxidants such as certain vitamins (e.g., vitamin C), polysaccharides, and other hydrophilic phytochemicals, which possess intrinsic antioxidant properties. These constituents are likely to play a significant role in the elevated AOA observed in the water extract. Concerning the relationship between antioxidants and GTG content, the data indicate that GTG contributes to the antioxidant activity; however, its effect may be either partial or synergistic with other bioactive compounds.

### 2.7. Relationship Between Molecular Orbitals and Antioxidant Activities of GTG and Established Antioxidant Molecules (Gallic Acid, Genistein, Quercetin, and Trolox)

The relationship between frontier orbital molecular energies and the antioxidant activities of compounds is fundamental to understanding their reactivity and effectiveness in mitigating oxidative stress. Key parameters include the energies of the highest occupied molecular orbital (HOMO) and the lowest unoccupied molecular orbital (LUMO), along with the HOMO–LUMO energy gap (ΔE). A smaller ΔE typically signifies a greater ease of electron transfer, which correlates with enhanced antioxidant activity due to increased electron-donating capacity. This mechanistic insight is essential for elucidating how antioxidants neutralize free radicals and for guiding the design of compounds with optimized activity through structural modifications. As summarized in Table 7, quercetin displayed the lowest ΔE (9.9001 eV), aligning with its superior antioxidant activity, evidenced by an IC_50_ of 5.99 ± 0.40 µM in the DPPH assay and a FeSO_4_/mM value of 1399.88 ± 16.96 µg/mL. Conversely, Trolox exhibited the highest ΔE (11.1993 eV), corresponding to the lowest activity, with an IC_50_ of 66.04 ± 0.22 µM and a FeSO_4_/mM value of 404.07 ± 8.94 µg/mL. The ΔE of GTG was comparable to that of genistein and gallic acid, ranging from 10.5581 to 10.6715 eV. Despite differences in IC_50_ values expressed in µg/mL, these compounds exhibited similar values in µM, spanning from 57.85 ± 2.00 to 64.57 ± 3.20 µM. Their FRAP values were 774.19 ± 10.49, 608.09 ± 17.70, and 623.32 ± 12.68 µg/mL of FeSO_4_/mM for gallic acid, genistein, and GTG, respectively. The inverse relationship between ΔE and antioxidant activity underscores the significance of frontier orbital energies in predicting the reactive potential of different compounds.

**Figure 8:**
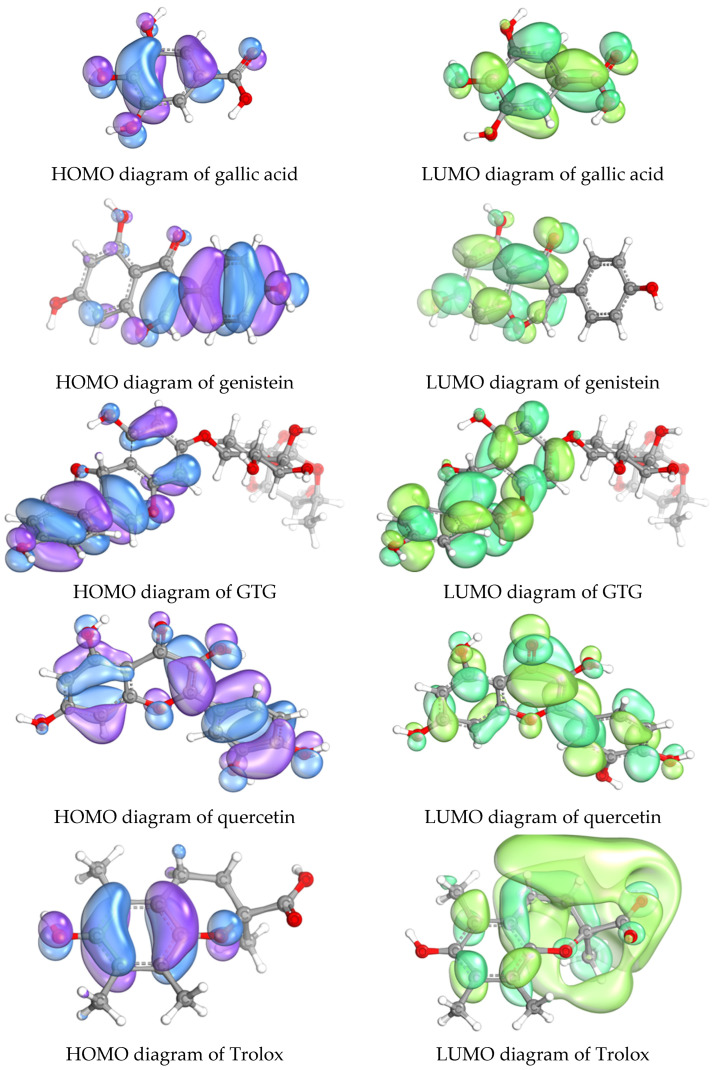
Diagrams depicting the frontier molecular orbitals of gallic acid, genistein, GTG, quercetin and Trolox, where the highest occupied molecular orbital (HOMO) is shown in violet/blue colors and the lowest unoccupied molecular orbital (LUMO) is shown in light green/dark green colors.

Genistein and GTG exhibited comparable antioxidant activities based on IC_50_ measurements in µM, primarily attributable to the aglycone component of genistein. As demonstrated in Figure 8, the frontier molecular orbital (FMO) diagrams for both genistein and GTG are centered on the aglycone moiety; the sugar portion of GTG did not display FMO diagrams, indicating that the antioxidant activity of GTG is predominantly governed by its aglycone structure. Additionally, the delocalization of unpaired electrons across conjugated systems—evident in quercetin and genistein—stabilizes the radicals formed during antioxidant action, thereby lowering their energy and enhancing radical scavenging capacity. Structural features such as conjugation and hydroxyl groups critically influence electronic properties and antioxidant activity by decreasing the HOMO-LUMO gap and promoting electron donation. Notably, these functional groups significantly contribute to the potent antioxidant activity observed in quercetin, where the FMO is delocalized across the entire molecule. This underscores the vital role of molecular architecture in modulating electronic and reactive properties pertinent to antioxidant efficacy.

## 3. Discussion

This study highlights the critical role of Hansen solubility parameters (HSP) and molecular orbital (MO) insights in optimizing the extraction and understanding the antioxidant activity of Genistein-7-O-α-rhamnopyranosyl-(1→6)-β-glucopyranoside (GTG) from *D. scandens*. The application of ultrasonic-assisted extraction (UAE) coupled with aqueous-ethanol solvents demonstrated that solvent composition significantly influences extraction efficiency, driven by the compatibility between solvent and solute as predicted by HSP. The analysis revealed that lower Ra values—particularly around 15.60 at 50% ethanol—correlate with higher GTG yields (~6.83 mg/g), illustrating that solvent systems with Hansen parameters closely matching those of GTG enhance solubility. Conversely, higher Ra values, such as 20.07 in pure ethanol, corresponded with markedly reduced yields, emphasizing the importance of solubility prediction models in selecting optimal solvents. The study also underscores the importance of solvent polarity and dielectric constant in maximizing extraction efficiency. The intermediate dielectric environment, achieved with 40–60% ethanol (dielectric constant ~50–60), facilitates hydrogen bonding with GTG’s hydroxyl groups, promoting solubilization. These findings aligned with previous research and indicate that polar solvents are generally more effective for the extraction of phenolic compounds. The Hansen solubility parameters provide a reliable framework for understanding solvent–solute interactions, confirming that solvents with suitable HSPs greatly enhance the extraction of phenolic compounds. Jeon et al. demonstrate that the HSP can effectively guide the selection of solvents for extracting compounds, emphasizing the influence of solvent compatibility on extraction efficiency [23].

In parallel, antioxidant activity assessments using DPPH and FRAP assays demonstrated that extracts obtained with polar solvents exhibited superior radical scavenging and reducing power. The highest activity was observed in water extracts, consistent with the higher solubility of phenolics and flavonoids, though no direct correlation between total phenolic content and antioxidant capacity was observed. A study by Mukit et al. examined the antioxidant activity of various extracts and found that chloroform and ethyl acetate extracts of *D. scandens* presented strong free radical scavenging activity as assessed by DPPH assay [2]. The application of HSP in the extraction of GTG from *D. scandens* facilitates the identification of suitable solvents capable of effectively dissolving specific chemical structures, thereby enhancing extraction efficiency and promoting sustainable processing. However, this approach does not account for the extraction of other phenolic acids and flavonoids. The lack of correlation between GTG content and measures such as TPC, TFC, and antioxidant activity suggests that there remain research gaps. Specifically, further studies are needed to explore additional phenolic acids, flavonoids, and other bioactive compounds with antioxidant properties present in *D. scandens*.

Molecular orbital studies reinforced the importance of the HOMO–LUMO gap (ΔE) in predicting antioxidant potential. Quercetin, with the lowest ΔE (9.9001 eV), exhibited the strongest activity, while Trolox’s higher ΔE (11.1993 eV) indicated lower reactivity. Similar trends were observed for genistein and gallic acid, confirming that molecules with lower ΔE are more effective antioxidants. These findings are consistent with prior literature, where flavonoids like quercetin demonstrated comparable or superior efficacy in radical scavenging. Analysis of FMO diagrams indicates that the antioxidant activity of GTG is primarily governed by the aglycone moiety. Conjugated systems and hydroxyl groups facilitate radical stabilization through electron delocalization, which reduces the HOMO–LUMO gap. A higher HOMO energy enhances electron donation to free radicals, thereby promoting radical neutralization, as discussed by Ozaki et al. [51]. Additionally, a smaller HOMO–LUMO gap is associated with increased antioxidant activity, as demonstrated by Farias et al. [36]. These findings suggest that structural features such as increased conjugation and hydroxylation can be targeted to optimize antioxidant efficacy. Hydroxyl groups, in particular, improve electron-donating capacity and stabilize radicals, further enhancing antioxidant performance, supported by studies from Cai et al. and Sarian et al. [52,53]. Overall, the electronic properties derived from FMOs provide critical insights into how structural modifications influence the antioxidant potential of GTG.

## 4. Materials and Methods

### 4.1. Materials

Plant materials were procured from a botanical shop in Bangkok, Thailand. The GTG standard was generously provided by Professor Dr. Waraporn Putalun from the Faculty of Pharmacy at Khon Kaen University, Thailand [43]. Chromatography-grade acetonitrile and methanol were obtained from Merck (Darmstadt, Germany). Formic acid, DPPH, and FeSO_4_ were supplied by Sigma-Aldrich Co. (St. Louis, MO, USA).

### 4.2. Calculation of Hansen Solubility Parameters

The Hansen solubility parameters (HSPs) for GTG were determined through the group contribution method described by Stefanis et al. [54], based on its molecular structure. The dispersion force (δ_d_), dipolar intermolecular force (δ_p_), and hydrogen bonding energy (δ_h_) values were calculated using Equations (1)–(3), respectively.

(1) $$\delta_{d}=\left(\sum_{i}N_{i}C_{i}+W\sum_{j}M_{j}D_{j}+17.3231\right){MPa}^{1/2}$$

(2) $$\delta_{p}=\left(\sum_{i}N_{i}C_{i}+W\sum_{j}M_{j}D_{j}+7.3548\right){MPa}^{1/2}$$

(3) $$\delta_{hb}=\left(\sum_{i}N_{i}C_{i}+W\sum_{j}M_{j}D_{j}W+7.9793\right){MPa}^{1/2}$$

In these equations, (C_i_) stands for the contribution of the first-order group of type (i), which occurs (N_i_) times within the target structure, while (D_j_) represents the contribution of the second-order group of type (j), appearing (M_j_) times. The constant (W) is assigned a value of 0 for compounds without second-order groups and 1 for those that contain them. Solvents with HSP values closely matching those of GTG were selected as extraction solvents. The compatibility between a solvent and the solute of interest is determined using the radius (Ra), calculated according to Equation (4).

(4) $$R_{a}^{2}=4{\left(\delta_{di}-\delta_{dj}\right)}^{2}+{\left(\delta_{pi}-\delta_{pj}\right)}^{2}+{\left(\delta_{hi}-\delta_{hj}\right)}^{2}$$

where i refers to the HSPs of the solute, and j refers to the HSPs of the solvent.

### 4.3. Preparation of Crude Extract

Ethanol, water, and a water–ethanol mixture were used as extraction solvents. The extraction procedure involved a solid–liquid extraction method, where 10 g of each sample was combined with the solvent at a ratio of 1:10 (*w*/*v*) and subjected to sonication for 30 min. After sonication, the mixture was filtered using a vacuum pump to separate the solvent from the solid residue. The solid residue was then re-extracted twice with fresh solvent. The obtained extracts were subsequently concentrated to dryness using a rotary evaporator. The dry weights of the extracts were recorded, and the samples were stored at −20 °C for further analyses.

### 4.4. HPLC Analysis of GTG in Derris scandens Extracts

#### 4.4.1. Identification of GTG by HPLC-MS

Chromatographic separation was conducted utilizing an Agilent Eclipse Plus C18 RRHD column (1.8 µm, 2.1 × 100 mm; Böblingen, Germany). The column compartment was maintained at a temperature of 25 °C, while the autosampler operated at 10 °C. The mobile phase was delivered at a flow rate of 0.5 mL/min, comprising 0.1% formic acid in ultrapure water (denoted as phase A) and 0.1% formic acid in acetonitrile (denoted as phase B). Sample pretreatment and analysis were executed under specific gradient conditions: the gradient began with 5% phase B for 5 min, increased to 40% phase B over a 5 min period, and then elevated to 70% phase B within an additional 5 min interval. This concentration of 70% of phase B was maintained for 5 min before returning to 5% phase B over the next 10 min, subsequently holding this concentration for an extra 10 min. The analysis was performed at a flow rate of 0.5 mL/min. The temperature of the column was maintained at 25 °C, and the injection volume was standardized at 5 µL. The LC system was coupled to an Agilent 6130 single quadrupole mass spectrometer (Agilent Technologies, Waldbronn, Germany) with an electrospray ionization (ESI) interface. Data were collected and analyzed by OpenLab CDS (Chromatography Data System) version 2.5, data acquisition and processing software (Agilent Technologies, Waldbronn, Germany). The LC-single quadrupole (SQ)-MS was used for all experiments and the measurements were operated in selected ion monitoring (SIM) acquisition mode. MS parameters included a capillary voltage of +3000 V, a drying gas flow of 3.0 L/min at 300 °C, and nebulizer pressure of 20 psi (nitrogen) were used for all experiments. Under the positive ionization mode (ESI+) and negative ionization mode (ESI−).

#### 4.4.2. Quantification of GTG in *Derris scandens* Extracts by HPLC-PDA

The HPLC system employed in this study was an Agilent 1260 Infinity II series (Santa Clara, CA, USA). This system included a quaternary pump, an autosampler, a multi-column thermostat, and a photodiode array detector. Chromatographic separation was performed using an ACE 5 C18-AR column (4.6 × 250 mm, 5 µm) obtained from Aberdeen, Scotland, UK, in conjunction with a Phenomenex C18 guard column (4 mm × 3 mm × 5 µm) from Torrance, CA, USA. The mobile phase comprised 0.1% formic acid in ultrapure water (designated as phase A) and 0.1% formic acid in acetonitrile (designated as phase B). The pretreatment sample was analyzed under a specific gradient protocol: beginning with a gradient increase from 15% to 25% phase B over 20 min, followed by an increase to 90% phase B over 5 min, maintaining this concentration for an additional 5 min, and concluding with a gradient return to 15% phase B over 3 min, held for 7 min. The analysis was conducted at a flow rate of 1 mL/min, with a detection wavelength set at 260 nm and the column temperature maintained at 25 °C, and the injection volume was set to 10 µL. The analytical method developed was subjected to both system suitability testing and method validation to ensure compliance with the standards established by the International Council for Harmonisation (ICH) for enhanced healthcare [47], as well as the AOAC Guideline 2016 [48].

### 4.5. Method Validation

The HPLC method was validated in accordance with AOAC guidelines, evaluating parameters including linearity, detection limit (LOD), quantification limit (LOQ), accuracy, and precision.

#### 4.5.1. Specificity

The specificity of the HPLC analysis was evaluated by comparing the chromatograms of the GTG standard solution with those of the sample solutions at a wavelength of 260 nm. It was demonstrated that the standard peak was clearly separated from other peaks in the sample chromatograms, as indicated by their respective retention times. Additionally, the specificity was further validated through three replicate analyses of both the standard and sample solutions.

#### 4.5.2. Linearity

Six-point calibration curves were developed using known concentrations of GTG spanning from 25 to 150 µg/mL, demonstrating a correlation coefficient exceeding 0.995 in accordance with AOAC guidelines. All measurements were conducted in triplicate.

#### 4.5.3. Limit of Detection (LOD) and Limit of Quantification (LOQ)

Using the data from the linear equations, the limit of detection (LOD) and limit of quantification (LOQ) were determined with the following formulas:

LOD = 3.3 × (Standard Deviation of Y-intercept)/(Mean of Slope)

LOQ = 10 × (Standard Deviation of Y-intercept)/(Mean of Slope)

The LOD and LOQ values were calculated as 3.3 and 10 times the ratio of the standard deviation of the calibration curve to the mean slope of the calibration curve, respectively.

#### 4.5.4. Accuracy

The recovery performance of the developed HPLC method was evaluated using spiked samples at three distinct concentration levels of GTG: 40, 80, and 120 µg/mL. Recovery percentages were calculated by comparing the measured concentrations to the actual known concentrations of the spiked samples. The method’s accuracy was confirmed through triplicate analyses.

#### 4.5.5. Precision

The method’s precision was evaluated by assessing repeatability through three consecutive injections within a single day at low, medium, and high concentration levels. To assess intermediate precision, three separate injections were performed on different days at the same concentration levels. The percentage relative standard deviation (%RSD) of the mean recovery percentage was calculated to quantify the method’s precision.

### 4.6. Preparation of In-House GTG

One gram of the crude water extracted from *D. scandens* was re-dissolved into 10 mL of water. Subsequently, the solution was partitioned with 10 mL of ethyl acetate in three separate phases to isolate non-polar compounds from the aqueous extract. The resulting fractions were then injected into semi-preparative HPLC for the separation and purification of GTG from the aqueous extract. The semi-preparative HPLC system employed in this study was an YL9100 series (YL Instrument Co, Yongin City, South Korea) using the Thermo Scientific^TM^ Hypersil^TM^ PREP C8 HPLC column (250 × 20 mm, 10 µm; Waltham, MA, USA). The mobile phase consisted of a 50% aqueous-methanol mixture. The flow rate was set to 5 mL/min, and detection was conducted at a wavelength of 260 nm with a total run time of 40 min. The crude extract, prepared at a concentration of 100 mg/mL, was injected into the HPLC system with an injection volume of 2 mL. The obtained GTG fractions were subsequently concentrated to dryness using a rotary evaporator and then identified by comparing their retention times and mass spectra with those of the GTG standard using LC-MS analysis.

### 4.7. Determination of Total Phenolic Content

The total phenolic content (TPC) was determined using the Folin–Ciocalteu method, with modifications tailored to the specific sample matrix. In this procedure, 1 mL of Folin–Ciocalteu reagent was mixed with 300 µg/mL of the sample extract and allowed to react for 5 min. Following this initial reaction, 1 mL of a 75 mg/mL sodium carbonate (Na_2_CO_3_) solution was added, and the final volume of the mixture was adjusted to 10 mL with distilled water. The reaction mixture was then incubated at room temperature for 90 min to facilitate complete color development. Subsequently, absorbance was measured at 765 nm against a blank. A standard curve for gallic acid was constructed using concentrations ranging from 10 to 40 µM, and TPC values were expressed as milligrams of gallic acid equivalents (GAE) per gram of dried original plant weight (mgGAE/g dried weight). Each sample was analyzed in triplicate to ensure statistical reliability.

### 4.8. Determination of Total Flavonoid Content

Total flavonoid content (TFC) was assessed using a modified colorimetric method. In this assay, 1 mL of an 8 mg/mL aluminum chloride (AlCl_3_) solution was added to 300 µg/mL of the sample extract, and the mixture was vortexed to ensure uniformity. Following a 1 h incubation period, absorbance was measured at 415 nm against a blank to determine flavonoid concentration. A calibration curve was established using quercetin standards at concentrations ranging from 15 to 80 µM. TFC values were expressed as milligrams of quercetin equivalents (QE) per gram of dried original plant weight (mgQE/g dried weight). All analyses were conducted in triplicate to ensure accuracy and consistency.

### 4.9. Evaluation of Antioxidant Activity

#### 4.9.1. DPPH Radical Scavenging Activity

The antioxidant activity of the extracts was initially evaluated using the DPPH (2,2-diphenyl-1-picrylhydrazyl) radical scavenging assay; 100 µL of the extract solution was combined with 100 µL of an ethanolic DPPH solution (0.6 mM) and vigorously mixed to ensure thorough interaction. The mixture was allowed to react for 30 min in a dark environment at room temperature. Following the incubation period, the absorbance of the resulting solutions was measured at 520 nm using a spectrophotometer, with a control sample consisting of the same DPPH concentration but without the extract.

Radical scavenging activity (RSA) was calculated as a percentage of DPPH discoloration using the following formula:

RSA (%) = [(Acontrol − Asample)/Acontrol] × 100%(5)

where Acontrol represents the absorbance of the DPPH in methanol, and Asample refers to the absorbance of the DPPH solution mixed with the plant extract. The IC_50_ values were determined through linear regression analysis to indicate antioxidant capacity. All samples were analyzed in triplicate to ensure reliability and accuracy.

#### 4.9.2. Ferric Reduction Ability-Antioxidant Power Test

For the Ferric Reducing Antioxidant Power (FRAP) test, the fresh working solution (FRAP reagent) included 300 mM acetate buffer (3.1 g C_2_H_3_NaO_2_·3H_2_O and 16 mL CH_3_COOH pH 3.6), 10 mM TPTZ (2,4,6-tripyridyl-s-triazine) solution in 40 mM HCl, and 20 mM FeCl_3_·6H_2_O solution were prepared. FeSO_4_ was used as a standard solution. Briefly, to some 100 µL of the sample solution (or standards at 1 mg/mL), 3 mL of the FRAP solution was added and mixed using a vortex, allowed to react in the dark at room temperature for 5 min. The absorbance measurement of the colored Fe-TPTZ complex was performed at 593 nm. Absorbance values were replaced in the FeSO_4_ standard curve equation (μg/mL). The results were expressed in microgram per milliliter equivalents of FeSO_4_ per gram of dry weight (µg/mL FeSO_4_/g DW).

### 4.10. Density Functional Theory (DFT) Calculations

This research employed Avogadro software (version 1.2.0) to generate the initial chemical structures [55]. Subsequently, the optimized xyz coordinates were obtained, and Orca input (.inp) files were prepared using these coordinates, applying the restricted Hartree–Fock (RHF) method with the def2-SVP basis set. Orca [56] was then executed on these input files: an administrator command prompt was opened, the directory was changed to the location of the .inp files, and assuming Orca was added to the system PATH variable, the command used was

Orca filename.inp > filename.out

From the same job folder, a Molden file was generated using the command:

Orca_2mkl filename-molden

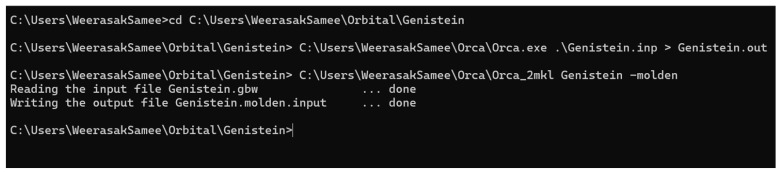



Finally, the Molden files were visualized with IboView software (version 20211019) [57], which was employed for intrinsic bond orbital (IBO) calculations, as well as for analyzing geometric structures and molecular orbital (MO) representations.

## 5. Conclusions

This study demonstrates that Hansen solubility parameters effectively guide the selection of optimal solvents for extracting phenolic compounds like GTG, with aqueous-ethanol mixtures around 40–60% yielding the highest extraction efficiency. The correlation between low Ra values and increased GTG content confirms the importance of solvent–solute compatibility and polarity, aligned with molecular orbital insights that elucidate antioxidant potential. MO analyses reveal that compounds with lower HOMO–LUMO gaps, such as quercetin, exhibit superior radical scavenging activity, emphasizing the role of molecular electronic properties in antioxidant efficacy. The findings suggest that polar solvents facilitate hydrogen bonding and solubilization of antioxidant constituents, enhancing biological activity. Overall, integrating HSP and MO approaches provides a comprehensive framework for optimizing extraction and understanding the mechanisms underlying antioxidant properties, facilitating the development of more effective phytochemicals with potential health benefits.

## Figures and Tables

**Table 1:** System suitability results of the analytical method.

Parameters	% RSD of Retention Time	% RSD of Peak Area	Resolution	Number of Theoretical Plate	Tailing Factor
Acceptance criteria	≤2%	≤2%	>2	2500	±1.2
Results	0.15	1.57	3.12	25,423	±1.01

**Table 2:** Method validation results of the analytical method.

Linearity	Linear Equation		R^2^		LOD (µg/mL)	LOQ (µg/mL)
	Y = 22.783X + 5.213		0.9999		0.39	1.18
	Y = 22.796X + 5.852		0.9999			
	Y = 22.697X + 0.906		0.9999			
mean	Y = 22.759X + 3.990		1			
Day	Concentration (µg/mL)		%Recovery	Mean recovery	SD	%RSD
	Actual	Found				
1	40	39.87	99.67	98.90	0.7417	0.75
	40	39.53	98.83			
	40	39.28	98.20			
	80	79.97	99.96	99.21	0.7175	0.72
	80	78.82	98.53			
	80	79.31	99.14			
	120	119.66	99.71	99.10	0.5719	0.58
	120	118.82	99.02			
	120	118.30	98.58			
2	40	39.94	99.85	98.61	1.4872	1.51
	40	39.61	99.02			
	40	38.78	96.96			
	80	79.68	99.59	99.68	0.6894	0.69
	80	79.22	99.03			
	80	80.32	100.40			
	120	118.31	98.59	98.46	0.6947	0.71
	120	118.90	99.09			
	120	117.26	97.71			
3	40	39.18	97.95	98.32	1.8939	1.93
	40	40.15	100.37			
	40	38.65	96.63			
	80	79.13	98.91	98.46	0.4691	0.48
	80	78.79	98.49			
	80	78.38	97.98			
	120	117.67	98.06	98.15	0.8601	0.88
	120	118.86	99.05			
	120	116.80	97.34			
Inter-Day (*n* = 9)	Concentration			Mean recovery	SD	%RSD
	40			98.61	1.2847	1.30
	80			99.11	0.7654	0.77
	120			98.57	0.7522	0.76

**Table 3:** Prediction of Hansen solubility parameters of GTG.

Group	Ni	Ci			(NiCi) d	(NiCi) *p*	(NiCi) h
1st-Order		δD	δP	δH			
CH3	1	−0.9714	−1.6448	−0.7813	−0.9714	−1.6448	−0.7813
CH2	1	−0.0269	−0.3045	−0.4119	−0.0269	−0.3045	−0.4119
CH	4	0.6450	0.6491	−0.2018	2.5800	2.5964	−0.8072
>CHOH	6	0.1123	0.2564	−0.1928	0.6738	1.5384	−1.1568
ACOH	2	0.5288	1.1010	6.9580	1.0576	2.2020	13.9160
AC	2	0.8446	0.6187	0.0084	1.6892	1.2374	0.0168
ACH	6	0.1105	−0.5303	−0.4305	0.6630	−3.1818	−2.5830
−CH=C<	1	0.5372	−0.9024	−1.8872	0.5372	−0.9024	−1.8872
>C=C<	1	0.3592	1.0526	−15.4659	0.3592	1.0526	−15.4659
>C=O	1	−0.4343	0.7905	1.8147	−0.4343	0.7905	1.8147
OH	4	−0.3462	1.1404	7.1908	−1.3848	4.5616	28.7632
O	5	0.0472	3.3432	0.9256	0.2360	16.7160	4.6280
			ΣNiCi		4.9786	24.6614	26.0454
**Group**	**Mj**		**Dj**		**(MiDi) d**	**(MiDi) *p***	**(MiDi) h**
**2nd-Order**		**δD**	**δP**	**δH**			
AC-O-C	2	0.2568	0.8153	0.6092	0.5136	1.6306	1.2184
ring of 6 carbon	2	−0.3874	−3.6432	0.0000	−0.7748	−7.2864	0.0000
ring of 5 carbon	3	−0.6681	−2.3430	−0.3079	−2.0043	−7.0290	−0.9237
cyclic-OH	2	−0.0876	−3.5220	0.5914	−0.1752	−7.0440	1.1828
			ΣMiDj		−2.4407	−19.7288	1.4775
ΣNiCi					4.9786	24.6614	26.0454
ΣMiDj					−2.4407	−19.7288	1.4775
Constant (C)					17.3231	7.3548	7.9793
ΣNiCi + ΣMiDj + C					19.8610	12.2874	35.5022

**Table 4:** Calculated radius (Ra) of HSP values of GTG with aqueous-ethanol solvents.

Name	Hansen Solubility Parameters			Ra
Solute	δD	δP	δH	
GTG	19.8610	12.2874	35.5022	
Solvent				
100% Ethanol	15.8	8.8	19.4	20.07
80% Ethanol	15.76	10.24	23.98	17.46
60% Ethanol	15.72	11.68	28.56	15.91
50% Ethanol	15.74	12.40	30.85	15.60
40% Ethanol	15.68	13.12	33.14	15.74
20% Ethanol	15.64	14.56	37.72	16.98
0% Ethanol	15.5	16	42.3	19.45

**Table 5:** Radius of Hansen solubility parameters (Ra), dielectric constants, and GTG content in various solvents (*n* = 3).

Solvents	Ra	Dielectric Constant (ε)	GTG Contents (mg/g Dried Weight) *
100% Ethanol	20.07	24.30	1.47 ± 0.02
80% Ethanol	17.46	37.70	5.17 ± 0.09
60% Ethanol	15.91	49.81	6.44 ± 0.09
50% Ethanol	15.60	55.42	6.83 ± 0.06
40% Ethanol	15.74	60.80	6.61 ± 0.08
20% Ethanol	16.98	70.82	5.26 ± 0.12
0% Ethanol	19.45	80.00	2.17 ± 0.11

* Significant differences between solvents (*p* < 0.05) were determined using a one-way ANOVA.

**Table 6:** Total phenolic content (TPC), total flavonoid content (TFC), and antioxidant activities (measured by DPPH and FRAP assays) of *Derris scandens* extracts (*n* = 3).

Sample	TPC * (mgGAE/g Dried Weight)	TFC * (mgQE/g Dried Weight)	DPPH * IC_50_ (mg/mL)	FRAP * (µg/mL FeSO_4_/g Dried Weight)
100% Ethanol extract	22.44 ± 2.73	44.25 ± 1.08	6.97 ± 0.08	253.04 ± 3.47
80% Ethanol extract	28.08 ± 5.57	32.81 ± 0.20	2.30 ± 0.02	422.16 ± 34.05
60% Ethanol extract	30.39 ± 0.50	25.63 ± 2.11	1.41 ± 0.01	495.49 ± 16.72
50% Ethanol extract	31.53 ± 0.61	21.21 ± 1.24	1.43 ± 0.03	513.97 ± 26.29
40% Ethanol extract	26.58 ± 3.78	14.58 ± 0.55	1.58 ± 0.03	550.32 ± 30.71
20% Ethanol extract	17.46 ± 1.65	12.42 ± 0.40	1.88 ± 0.01	460.56 ± 29.50
0% Ethanol extract	17.99 ± 2.96	22.33 ± 1.77	1.08 ± 0.01	762.46 ± 47.95

* Significant differences between solvents (*p* < 0.05) were determined using a one-way ANOVA.

**Table 7:** Antioxidant activities and calculated HOMO and LUMO energies (in electron volts, eV) of gallic acid, genistein, GTG, quercetin, and Trolox.

Compounds	M.W.	HOMO (eV)	LUMO (eV)	ΔE (eV)	DPPH (IC_50_)		FRAP FeSO_4_/mM (µg/mL)
					µg/mL	µM	
Gallic acid	170	−8.3543	2.2706	10.6249	10.54 ± 0.35	62.00 ± 2.06	774.19 ± 10.49
Genistein	270	−7.7018	2.8563	10.5581	15.62 ± 0.54	57.85 ± 2.00	608.09 ± 17.70
GTG	578	−7.9859	2.6856	10.6715	37.32 ± 1.85	64.57 ± 3.20	623.32 ± 12.68
Quercetin	302	−7.8091	2.0910	9.9001	1.81 ± 0.12	5.99 ± 0.40	1399.88 ± 16.96
Trolox	250	−7.5032	3.6961	11.1993	16.51 ± 0.22	66.04 ± 0.22	404.07 ± 8.94

## Data Availability

Data is contained within the article.

## Abbreviations

The following abbreviations are used in this manuscript:

AlCl_3_	Aluminum chloride
AOA	Antioxidant activity
AOAC	Association of Official Analytical Collaboration
°C	Celsius degree
cm	Centimeter
Da	Dalton
DFT	Density functional theory
DPPH	2,2-Diphenyl-1-picrylhydrazyl
FRAP	Ferric reducing antioxidant power
eV	Electron volt
ESI	Electrospray ionization
FeSO_4_	Ferrous sulfate
FMO	Frontier molecular orbital
g	Gram
GAE	Gallic acid equivalent
GC-MS	Gas chromatography-mass spectrometry
GTG	Genistein-7-O-[α-rhamnopyranosyl-(1→6)]-β-glucopyranoside
HCl	Hydrochloric acid
HOMO	Highest occupied molecular orbital
HPLC	High Performance Liquid Chromatography
HSP	Hansen Solubility Parameters
IBO	Intrinsic bond orbital
IC_50_	Half–maximal inhibitory concentration
ICH	The International Council for Harmonisation of Technical Requirements for Pharmaceuticals for Human Use
LUMO	Lowest unoccupied molecular orbital
L	Litter
LOD	Limit of Detection
LOQ	Limit of Quantitation
mL	Milliliter
mM	Millimolar
Mol	Mole
MW	Molecular weight
Na_2_CO_3_	Sodium carbonate
QE	Quercetin equivalent
Ra	Hansen solubility parameter distance
SD	Standard deviation
TFC	Total flavonoid content
TPC	Total phenolic content
TPTZ	2,4,6-tripyridyl-s-triazine
UAE	Ultrasonic-assisted extraction
UV	Ultraviolet
δD	Dispersion force
δH	Hydrogen bonding energy
δP	Dipolar intermolecular force
µg	Micro gram
µM	Micro molar
%RSD	Percentage relative standard deviation
